# Do Elevated YKL-40 Levels Drive the Immunosuppressive Tumor Microenvironment in Colorectal Cancer? Assessment of the Association of the Expression of YKL-40, MMP-8, IL17A, and PD-L1 with Coexisting Type 2 Diabetes, Obesity, and Active Smoking

**DOI:** 10.3390/cimb45040182

**Published:** 2023-03-27

**Authors:** Błażej Ochman, Sylwia Mielcarska, Agnieszka Kula, Miriam Dawidowicz, Julia Robotycka, Jerzy Piecuch, Monika Szrot, Sylwia Dzięgielewska-Gęsiak, Małgorzata Muc-Wierzgoń, Dariusz Waniczek, Elżbieta Świętochowska

**Affiliations:** 1 Department of Medical and Molecular Biology, Faculty of Medical Sciences in Zabrze, Medical University of Silesia, 19 Jordana, 41-800 Zabrze, Poland; d201109@365.sum.edu.pl (S.M.); s78516@365.sum.edu.pl (J.R.); eswietochowska@sum.edu.pl (E.Ś.); 2 Department of Oncological Surgery, Faculty of Medical Sciences in Zabrze, Medical University of Silesia, 35 Ceglana, 40-514 Katowice, Poland; d201070@365.sum.edu.pl (A.K.); d201069@365.sum.edu.pl (M.D.); dwaniczek@sum.edu.pl (D.W.); 3 Department of General and Bariatric Surgery and Emergency Medicine in Zabrze, Faculty of Medical Sciences in Zabrze, Medical University of Silesia, 10 Marii Curie-Skłodowskiej, 41-800 Zabrze, Poland; jerzypiecuch@sum.edu.pl (J.P.); mszrot@sum.edu.pl (M.S.); 4 Department of Internal Medicine Prevention in Bytom, Medical University of Silesia, 41-808 Katowice, Poland

**Keywords:** YKL-40 (CHI3L1), PD-L1, colorectal cancer

## Abstract

The influence of chitinase-3-like protein 1 (YKL-40 or CHI3L1) expression on the immunological properties of the tumor microenvironment, which may affect the effectiveness of immunotherapy, is currently not sufficiently understood in colorectal cancer (CRC). The aim of this study was to investigate the relationship between YKL-40 expression and the immunological properties of the tumor microenvironment in CRC. We performed in silico analysis, including analysis of immune cell infiltration scores and the immune landscape depending on YKL-40 expression, gene set enrichment analysis (GSEA), and analysis of three Gene Expression Omnibus (GEO) datasets. In 48 CRC tissue homogenates and the surgical margin, we analyzed the expression of YKL-40, MMP8, IL17A, and PD-L1. Moreover, we analyzed the expression of YKL-40 in tissue homogenates retrieved from patients with coexisting diabetes, obesity, and smoking. The expression of YKL-40 was significantly higher in CRC tumor tissue compared to healthy tissue and correlated with MMP-8, IL17A, and PD-L1 expression. In silico analysis revealed an association of YKL-40 with disease recurrence, and GSEA revealed a potential link between elevated YKL-40 expression and immunosuppressive properties of the tumor microenvironment in CRC.

## 1. Introduction

One of the most important factors determining prognosis in colorectal cancer (CRC) is the presence of metastases. Since metastatic CRC exhibits a poor prognosis, even with complete resection of the primary tumor, identification of new effective therapeutic targets to disrupt pathways involved in different stages of CRC metastasis is urgently needed [1]. Intercellular interactions in an abnormal tumor environment consisting of cancer and non-cancer cells, blood vessels, stroma, and the extracellular matrix might contribute to the high heterogeneity of CRC. For that reason, the significance of the tumor microenvironment (TME) in modulating CRC progression and metastasis has been extensively investigated in recent years. Cytokines, immune checkpoints, and proteins involved in extracellular matrix remodeling such as metalloproteinases and chitinases are among numerous molecules that shape the TME to support tumor growth and progression and immune evasion.

Chitinase-3-like protein 1 (CHI3L1), also known as YKL-40, is a glycoprotein and a member of the 18-glycosyl hydrolase family with no chitinase activity [2,3]. YKL-40 is expressed in numerous types of cells, including macrophages, neutrophils, vascular smooth cells, chondrocytes, epithelial cells, and synoviocytes [2,4,5]. High levels of this glycoprotein have also been found in several tumors, such as colon, ovary, prostate, breast, lung, and kidney cancers, as well as melanoma. YKL-40 is closely related to chronic inflammation, cancer, and increased tissue remodeling [1,2]. Increased expression of YKL-40 was observed a the group of patients with obesity and type 2 diabetes, probably due to the involvement of YKL-40 in inflammatory processes [3,4]. It has been proven that YKL-40 promotes tumor progression, mainly through the regulation of extracellular matrix remodeling and inflammation. Many studies indicate that its serum level is associated with adverse clinical outcomes of patients with CRC and other solid tumors and might serve as a marker of poor prognosis [1,5].

IL-17 is a proinflammatory cytokine that has been shown to increase tumor growth, invasion, metastasis, and angiogenesis [6,7]. It is produced by neutrophils, macrophages, dendritic cells, and CD8+ T cells; however, the major source of IL-17 is the Th17 cells present in the TME [6,8,9]. The interplay between IL-17 and IL-23, by reducing the number of CD8+ T cells; increasing the activity of Treg cells; and modulating MMP-9, MMP-7, MMP-2, VEGF, TGF-β, IL-6, and CCR6 expression; has a crucial role in the pathogenesis of CRC [10,11,12,13,14]. A high intratumoral level of IL-17 is associated with unfavorable outcomes in CRC [6].

The PD-1/PD-L1 pathway induces downregulation of T-cell activity and inhibits the release of cytokines, thereby providing the immune tolerance to self-antigens [15,16]. The upregulation of PD-L1, which is observed in multiple types of cancer, appears to be one of the major mechanisms allowing tumor cells to evade immune surveillance and avoid immune attack [17,18]. It has also been shown that PD-L1 can bind to appropriate RNAs and protect target RNAs from degradation by RNA exosome components. The relationship between PD-L1 and the stability of the mRNA of genes involved in the process of cancer progression, such as *NBS1* and *BRCA1*, seems to affect the effectiveness of radiation and chemotherapy; therefore, targeting PD-L1 may increase sensitivity to chemotherapies and radiotherapy [19]. Targeting the PD-1/PD-L1 axis is the aim of many immunotherapeutic strategies used in the treatment of various tumors, which have developed rapidly in recent years [20]. Immune checkpoint inhibitors (ICIs), especially anti-PD-1 therapy, have dramatically reshaped the landscape of cancer therapy; however, the use of ICIs in CRC is limited to microsatellite instability-high (MSI-H) tumors, which are found in approximately 15% of CRC cases. The search for new molecules involved in pathways related to CRC progression and metastasis has been extensively evaluated, which may result in the development of new targeted therapy options. Immunotherapy has been revealed as more effective than conventional cytotoxic therapy in many cases, achieving long-term benefits in a subset of patients with advanced disease [21]. However, due to the emerging therapeutic resistance to PD-1/PD-L1 axis inhibitors, there is a need for different strategies to target the PD-1/PD-L1 axis or alternative strategies to overcome acquired resistance [22,23]. Recent reports on the relationship between the expression, stability, and activity of PD-L1 in cancer cells and factors regulating the cytoplasmic domain of PD-L1 (PD-L1-CD), such as acidic phospholipids, electrostatic interaction with the plasma membrane [24] and the effect of AMP-activated protein kinase (AMPK) and metformin on PD-L1 levels [25], together with the increasingly accurate understanding of the function of mitochondria and the development of selective nanoparticles targeting the mitochondrial PD-L1 regulatory mechanisms [26,27], may contribute to a breakthrough in the effectiveness of methods targeting the PD-1/PD-L1 axis and in photodynamic therapy, in which PD-L1 expression induced by interferon gamma is currently one of the greatest limitations to the use of this method in cancer treatment [27,28,29].

YKL-40 has been reported to be involved in immune processes in the tumor microenvironment. However, its exact role and connection with immune molecules still require deeper investigation. The aim of this study is to investigate the associations between YKL-40 and selected factors in the context of the modulation of immune and tissue remodeling processes in colorectal cancer. We analyzed the expression of YKL-40, MMP-8, IL-17A, and PD-L1 in three Gene Expression Omnibus (GEO) datasets. We performed gene set enrichment analysis (GSEA) and CIBERSORT score analysis based on the expression of YKL-40 in CRC tumors. Furthermore, we measured levels of YKL-40, MMP-8, IL-17A, and PD-L1 in the tissue homogenates obtained from CRC tumors. These data were compared with the clinicopathological features of the patients.

## 2. Materials and Methods

### 2.1. Analysis of Immune Cell Infiltration Scores and the Immune Landscape and Gene Set Enrichment Analysis (GSEA) in Colorectal Cancer

To estimate the abundance of immune cell types under high and low YKL-40 expression and to evaluate the corresponding intratumoral immune cell composition, we used the CAMOIP tool [30]. Analysis of immune cell infiltration scores and the immune landscape was performed on ‘TCGA-COAD’.

GSEA was performed using Tumor Cancer Genome Atlas (TCGA) RNA-seq data for CRC retrieved from the package FieldEffectCRC [31] in R studio. GSEA was conducted among cohort A, consisting of 311 CRC samples. We normalized the matrix data using the DESeq2 package [32]. Then, we divided the cohort into high versus low expression of YKL-40. Further analysis was performed using the package “fgsea” in R studio. The corresponding gene set for pathways and biological processes “H: hallmark gene sets h.7.5.” was obtained from Human MSigDB Collections [33,34].

The genes with significant differences in expression among high vs. low YKL-40 (CHI3L1) expression were screened for GO enrichment analyses (|logFC| > 0.5 and p.adj. < 0.05).

### 2.2. Gene Expression Omnibus (GEO) Dataset Analysis

Datasets containing array-based normalized mRNA expression profile data were obtained from the Gene Expression Omnibus (GEO) platform. Analysis of 136 samples of CRC and non-cancerous colonic tissue obtained from GEO datasets: GDS4382 [35], GDS4513 [36], and GDS4515 [37] was conducted to predict and later validate expression and correlation between YKL-40, MMP8, IL-17A, and PD-L1. The GDS4515 dataset provided expression data of the mentioned proteins in 34 MSI CRC tumors and 15 normal colonic mucosa samples. GDS4513 included YKL-40 expression in tumor cells obtained from sporadic stage UICC II CRC patients treated with standard oncological resection who either relapsed or did not relapse during the 5-year follow-up.

### 2.3. Tissue Homogenates of CRC

The study involved 48 specimens of colorectal cancer tumor tissue and surgical margin tissue collected from 20 female and 28 male patients who had undergone surgeries in the 1st Specialistic Hospital in Bytom, Poland (Research Ethics Committee approval no. KNW/0022/KB1/42/III/14/16/18, 14.07.2020). Inclusion criteria involved the confirmation of colorectal adenocarcinoma and negative surgical margin without tumor infiltration confirmed in a histological examination, patient age > 18 years old, and a signed written consent. Patients with tumor types other than adenocarcinoma, the presence of margin infiltration, a lack of signed consent to participate in the study, age < 18 years old, and with a history of chemo- or radiotherapy were excluded from the study. The tumor stage was classified using the TNM staging system and grading. Patient data on diabetes, smoking, obesity, and BMI were included. Patients with a BMI of 30.0 or higher were considered obese. The smoking group included active smokers, and non-active smokers were included to the non-smoking group. The characteristics of the study group are presented in Table 1.

### 2.4. Preparation of Samples

Colorectal cancer tumor tissues and surgical margins from the respective patients were weighed and homogenized in a phosphate buffer environment. Homogenization was performed using a PRO 200 homogenizer (PRO Scientific Inc., Oxford, CT, USA) at 10,000 rpm. Nine volumes of phosphate-buffered saline were added to each tumor tissue and the surgical margin. Subsequently, the obtained suspensions were sonicated using an ultrasonic cell disrupter (UP100, Hilscher, Germany). The tissue homogenates obtained in this way were centrifuged at 12,000 rpm for 15 min at 4 °C. Total protein concentration was measured by colorimetry using a reagent kit with the pyrogallol-red method (Sentinel Diagnostics, Milano, Italy). The results were read using a 600 nm light wavelength at 37 degrees with a Technicon RA-XTTM biochemical analyzer (Technicon Instruments Corporation, Mahopac, NY, USA).

### 2.5. Evaluation of YKL-40, MMP8, IL17A, and PD-L1 Levels

The concentrations of the examined proteins in the tissue homogenates were determined using appropriate enzyme-linked immunosorbent assay (ELISA) kits. CHI3L1 (YKL-40) concentration was measured using a MICROVUE YKL-40 ELISA kit (Quidel, San Diego, CA, USA). The limit of detection (LOD) for the used YKL-40 ELISA kit was 5.4 ng/mL. A human matrix metalloproteinase-8 ELISA kit (Biovendor, Brno, Czech Republic) with a minimum detectable concentration 0.025 ng/mL was used to measure MMP-8 concentration. The concentration of IL-17A was assessed using a human IL-17A ELISA kit (Diaclone, France) with a sensitivity of 2.3 pg/mL. A human PD-L1 ELISA kit (Cloud Clone, Wuhan, China) with an LOD of 0.057 ng/mL was used to measure the concentration of PD-L1 in tissue homogenates. The absorbance of samples was measured at a wavelength of 450 nm using a universal microplate spectrophotometer (μQUANT, Biotek Inc., Charlotte, VT, USA). The obtained results were recalculated relative to the corresponding total protein level and presented as ng/mg of protein.

### 2.6. Statistical Analysis

Statistical analysis of the data included the log transformation of the obtained data, examination of the distribution of data, differences in protein concentration in the tumor, and tissue margin groups, as well as examination of the correlations. Log transformation of the levels of the examined molecules provided a better fit to the Gaussian distribution. Data are presented as mean ± SD for the variables with normal distribution and as median with interquartile range for the variables with non-normal distribution. A paired Student’s t-test and the Mann–Whitney U test were used to compare levels of examined proteins in tumor tissues and tissue margins. We used Pearson’s coefficient to investigate the correlation between the tested proteins. Kendalls Tau rank correlation coefficient was used to determine the association between the levels of the examined proteins, T, and N parameters. Statistical analysis was performed using STATISTICA 13 software (Statsoft) and the ggplot2-R package dedicated to data visualization in RStudio software (Integrated Development for R. RStudio, PBC, Boston, MA, USA).

## 3. Results

### 3.1. GEO Profile Analysis

To predict and validate the interactions of YKL-40 in the tumor microenvironment, we analyzed mRNA expression of YKL-40, IL-17A, PD-L1, and MMP-8 in 136 samples of CRC tissue and non-cancerous colonic tissue obtained from Gene Expression Omnibus (GEO) datasets GDS4382, GDS4513, and GDS4515.

**Figure 1:**
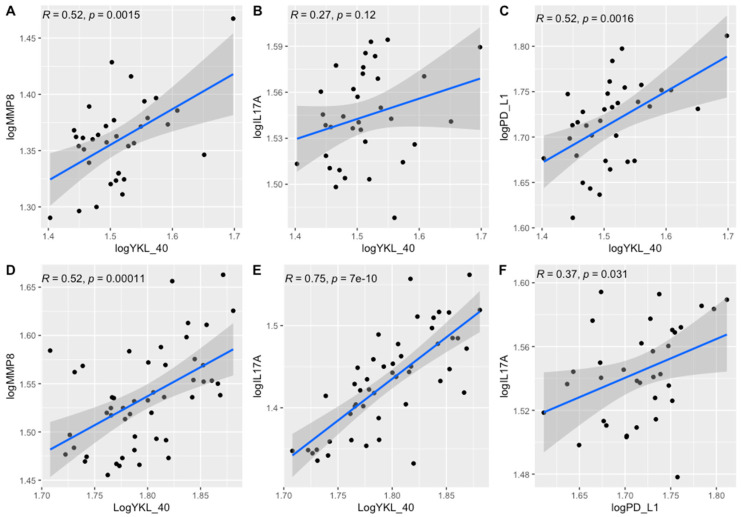
Correlations between the expression of YKL-40, MMP8, PD-L1, and IL17A in GEO datasets GDS4382 (**A**–**C**,**F**) and GDS4515 (**D**,**E**).

We observed positive correlations between the expression of YKL-40, IL-17A, PD-L1, and MMP-8 (Figure 1) in GEO dataset GDS4382, containing 34 CRC tumors and adjacent non-cancerous tissues (Figure 1A–C,F), and in GEO dataset GDS4515, with 49 CRC microsatellite instable tumors and normal colonic mucosa samples (Figure 1D,E).

**Figure 2:**
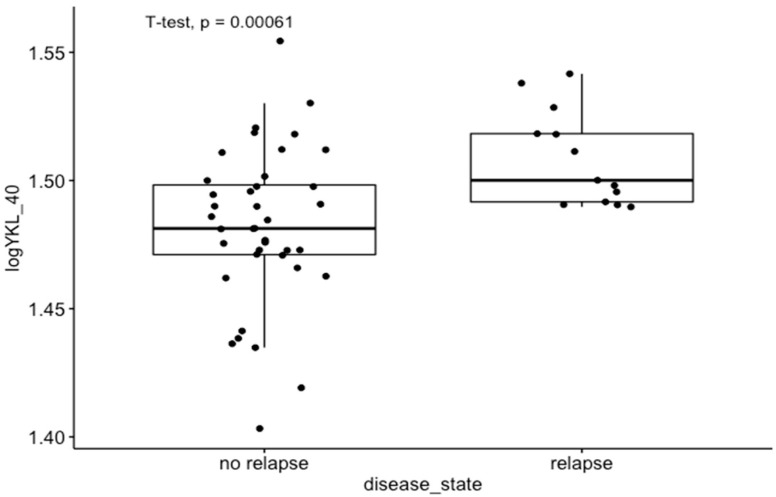
Differences in YKL-40 expression levels between relapse and non-relapse groups of CRC patients in GEO dataset GDS4513.

Moreover, in GEO dataset GDS4513, which was analyzed for molecular comparison between the relapse and non-relapse groups of CRC patients, we observed that YKL-40 expression was significantly higher in the CRC relapse group than in the CRC non-relapse group (*p* < 0.01) (Figure 2). A complete 5-year follow-up was available for 53 patients [34].

### 3.2. GSEA Results: Predicted Pathways and Biological Processes for High–Low YKL-40 Expression

**Figure 3:**
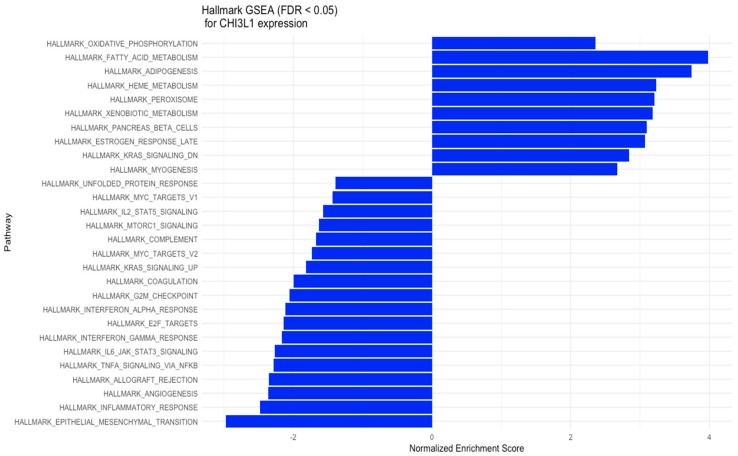
GSEA for the expression of YKL-40 (CHI3L1) in various hallmark gene sets.

The results of the GSEA analysis for YKL-40 expression were read for pathways from hallmark gene sets of the MSigDB Collection (Figure 3). The hallmark gene sets with positive NES scores (NES > 2) for YKL-40 (CHI3L1) expression include oxidative phosphorylation, fatty acid metabolism, peroxisomes, heme metabolism, and downregulation of the KRAS signaling pathway, while the hallmark gene sets with negative NES scores (NES < −1) for YKL-40 expression include Myc targets, IL-2 STAT5 signaling, mTORC1 signaling, complement, G2M checkpoint, interferon alpha response, E2F targets, interferon gamma response, IL6-JAK-STAT6 signaling, TNF-alpha signaling via NFKB, allograft rejection, angiogenesis, and KRASsignaling upregulation (Figure 3).

### 3.3. Exploration of Immunogenicty and the Immune Infiltration Landscape of YKL-40 Based on TCGA-COAD Data

**Figure 4:**
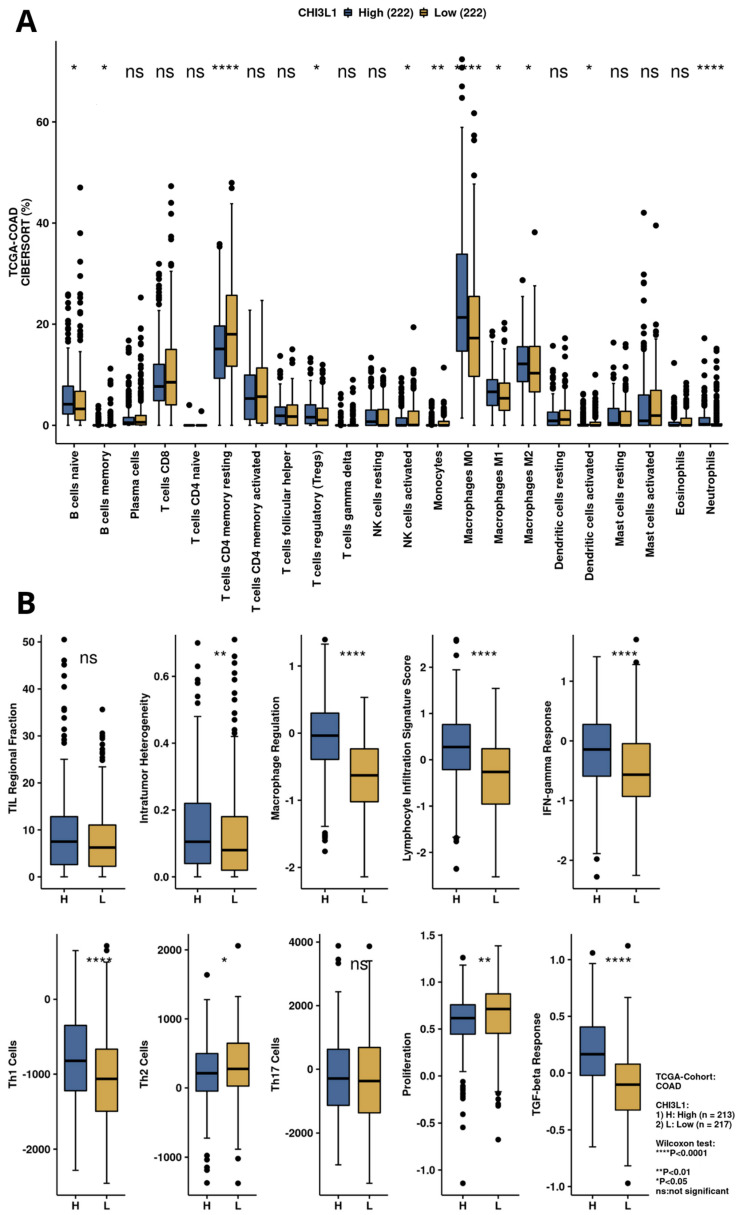
Immune infiltration and immunogenicity analysis for YKL-40 (CHI3L1) based on TCGA-COAD data. (**A**) Infiltration of immune cells for CHI3L1 expression groups calculated by CIBERSORT algorithm. (**B**) Immune-related scores for high and low CHI3L1 expression groups.

The immune scores of intratumor heterogeneity, macrophage regulation, lymphocyte infiltration signature score, IFN-gamma response, Th1 cells, and TGF-beta response were significantly upregulated in the YKL-40 high-expression group (Figure 4A,B), unlike proliferation and the Th2 cell score, which were decreased in this group. Moreover, CIBERSORT analysis revealed elevated percentages in the tumor microenvironment of naïve B cells; Tregs; macrophages M0, M1, and M2; and neutrophils in the YKL-40 high-expression group, whereas in the YKL-40 low-expression group, elevated percentages of resting T-cell CD4 memory and monocytes were observed.

### 3.4. Results Obtained from the Tissue Homogenates

**Figure 5:**
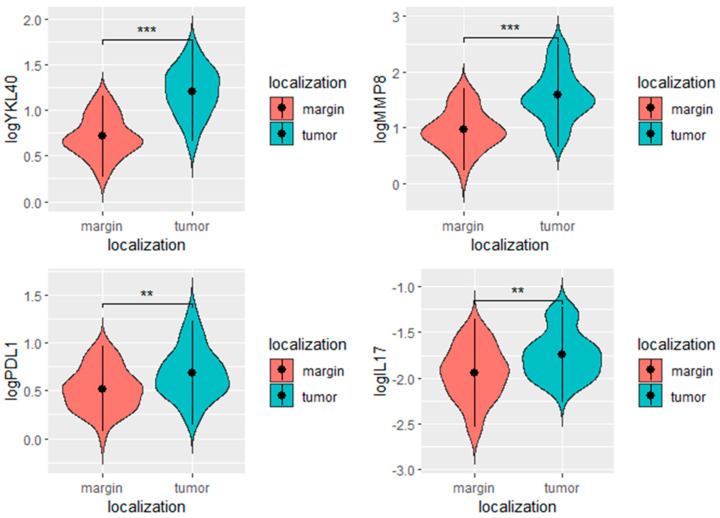
YKL-40, PD-L1, MMP-8, and IL-17 levels in the tumor and margin tissues. *** *p* < 0.001, ** *p* < 0.01, ns- not significant.

We found significantly higher levels of YKL-40, PD-L1, MMP8, and IL-17 in the tumor in comparison to the margin tissue (Table 2, Figure 5).

**Figure 6:**
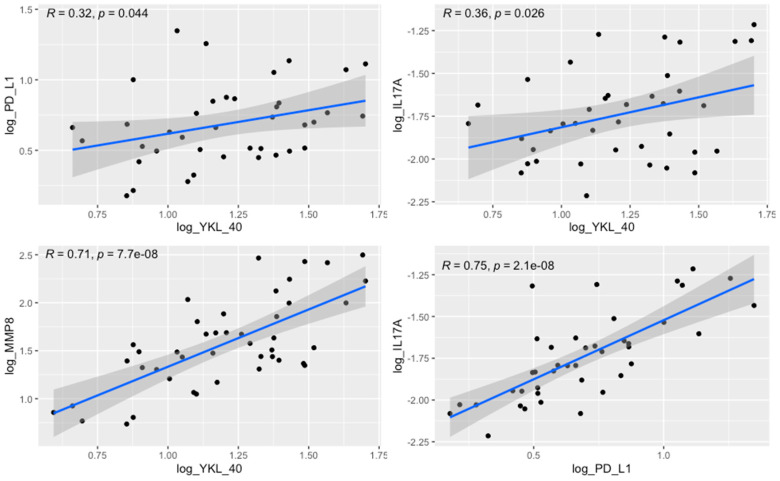
Correlation between the tumor levels of YKL-40 and PD-L1 (R = 0.32, *p* = 0.044), YKL-40 and IL-17 (R = 0.36, *p* = 0.026), YKL-40 and MMP-8 (R = 0.71, *p* < 0.0001), and PD-L1 and IL-17 (R = 0.75, *p* < 0.0001).

**Figure 7:**
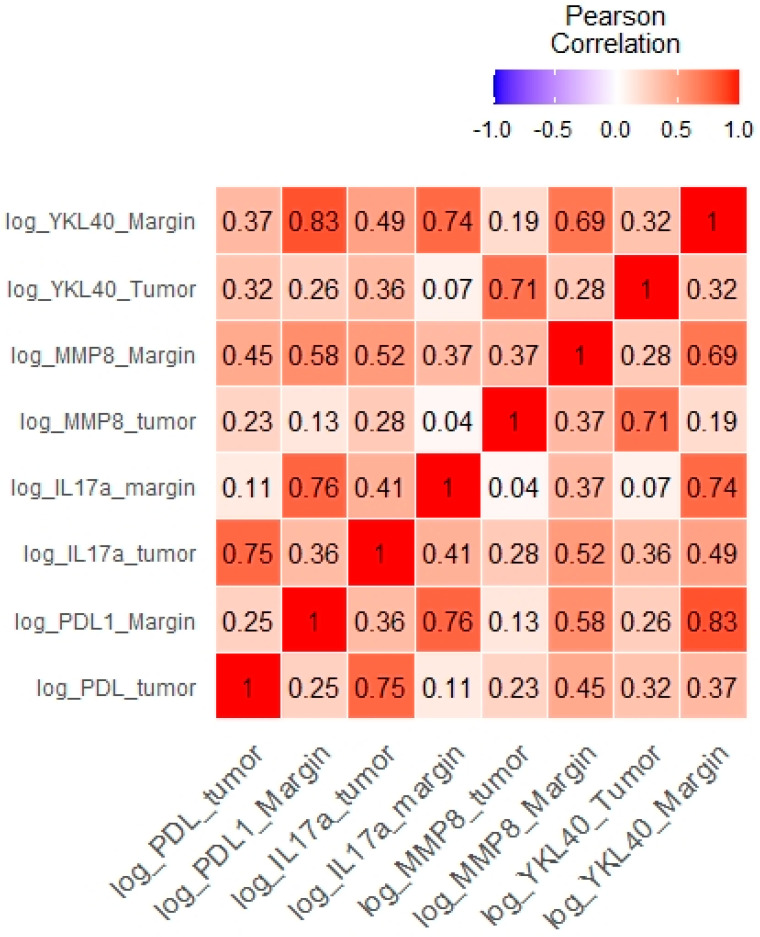
Correlations between the levels of the examined molecules presented as a heat map.

Furthermore, the tumor levels of YKL-40 correlated significantly with those of MMP-8, IL-17, and PD-L1 levels. The margin levels of YKL-40 were significantly associated with the margin levels of MMP-8, IL-17, and PD-L1 (Table 3, Figure 6). The results of other correlations between the examined molecules are presented in Figure 7. Results obtained from tissue homogenates are in line with in silico analysis of GEO datasets.

We did not observe any associations between the concentrations of the investigated molecules and the occurrence of diabetes, obesity, and smoking among patients (Table 4).

## 4. Discussion

YKL-40 was found to play a pivotal role in cancer through its involvement in tissue remodeling and inflammation in the tumor microenvironment. At the molecular level, YKL-40, through its functional heparin-binding domain, has the potential to interact with heparan sulfate (HS) fragments, heparin, and other glycosaminoglycans such as hyaluronan [38,39,40]. YKL-40 binding to collagen types I, II, and III was also reported [41].

Although specific receptors for YKL-40, such as CD44 [42], galectin-3 (Gal-3) [43], and interleukin-13 receptor subunit alpha-2 (IL-13Rα2) [44], have been identified, the heparin-binding motif seems to be crucial for YKL-40 activity. The main source of cell HS is syndecan-1, a transmembrane molecule with cytoplasmic domains that can interact with several regulators and act as a coreceptor with adjacent transmembrane receptors, such as integrins [42]. Altered interaction of syndecan-1 and integrin αv β3 caused by YKL-40 can affect cell adhesion provided by integrins, increasing the metastatic potential of cancer cells. Consistent with available data, the interaction of syndecan-1 with integrins induces phosphorylation of the adhesive kinase (FAK) and stimulation of MAPK8 [45]. These signaling pathways have been reported to support tumor growth and progression by inducing angiogenesis, increasing cancer cell motility and invasion, and promoting EMT [46,47]. Additionally, YKL-40 was found to stimulate the expression of C-chemokine ligand 2 (CCL2) and chemokine CX motif ligand 2 (CXCL2) by activating ERK1/2 and JNK signaling pathways [48]. CCL2 and CXCL2 have a chemotactic effect on macrophages and neutrophils, enhancing the infiltration of immune cells in the TME. Thus, we may suspect that YKL-40 secreted into extracellular space by tumor cells, macrophages, and neutrophils has the potential to promote extracellular matrix remodeling through interactions with TME elements and to support tumor growth by inducing activation of signaling pathways involved in cell survival and the EMT. This is in line with the CIBERNET results of the increased proportion of relevant subpopulations of immune cells, such as neutrophils, and macrophages in CRC tissues with high expression of YKL-40 (Figure 4). Neutrophils infiltrating the tumor microenvironment contribute to the increased migration of cancer cells by destroying the extracellular matrix components and can drive the EMT process [49,50]. Tumor-associated macrophages (TAMs), by secreting many factors, such as TGF-B, TNF-a, IL-1B, and IL-6, may be responsible for inducing the EMT process in CRC [51,52,53,54]. However, GSEA results revealed that the genes involved in the EMT pathway are mostly downregulated in colorectal explants with elevated YKL-40 expression (Figure 3). In GEO analysis (Figure 2), we observed significantly elevated YKL-40 expression in the group with disease recurrence and a significant correlation between YKL-40 and MMP-8 levels (Figure 1 and Figure 6).

Both analysis of GEO datasets and results obtained from tissue homogenates demonstrated higher expression of YKL-40 and MMP-8 in tumor tissue compared with normal colon mucosa and a positive association between YKL-40 and MMP-8 expression in the tumor. MMP-8 was reported to be associated with distant metastasis, inflammation, poor survival, and CRC progression, but it is also considered to play a protective role in the development of some malignancies due to its antitumor activity [55,56,57]. However, whether MMP-8 expression could be directly upregulated by YKL-40 requires deeper investigation.

YKL-40 was observed to be associated with the PI3K/AKT/mTOR pathway, in which aberrant activation plays an important role in the development of early stages of colon cancer. A close interplay between PI3K/AKT and Wnt/β-catenin signaling pathways has been described in CRC; inhibition of one of the pathways causes hyperactivation of the other pathway. Both pathways are associated with the regulation of multiple cellular events in CRC, including the induction of the expression of proinflammatory cytokines, such as IL-17A and TGF-B1. TGF-B1 expression has been suggested to be associated with YKL-40 expression, mediating many pathways involved in tumor progression and, importantly, acts together with IL-23 during the differentiation of Th17 cells, which are the main source of IL-17. In the GEO datasets and the tissue homogenates, we found a positive correlation between the tumor concentrations of YKL-40 and IL-17a (Figure 1 and Figure 6). According to previous studies, the interaction of YKL-40 with Gal-3 [45] and interactions of IL-13RA2-YKL-40 complexes with THEM219 [46] could augment IL-17A expression by activation of MAPK/Erk and Wnt/β-catenin signaling pathways. Serum levels of YKL-40 were reported to correlate with those of IL-17 in patients with psoriasis, but no data are available regarding CRC patients [58].

The interplay between YKL-40, IL-17A, and PD-L1 appears to be worthy of investigation in terms of CRC immunotherapy. Liu et al. showed a significant regulatory effect of IL-17A on PD-L1; IL-17A causes increased expression of PD-L1 through the p65/NRF1/miR-15b-5p pathway and promotes resistance to anti-PD-1 therapy [59]. Our in silico analysis demonstrated interaction between PD-L1 and IL-17, especially in GEO dataset containing CRC MSI-high tumors; similarly, data obtained from our tissue homogenates of CRC confirmed a positive correlation between tumor concentrations of PD-L1 and IL-17. These findings seem to confirm crosstalk between PD-L1 and IL-17 pathways and support the literature data. In CRC, the expression of YKL-40 was reported to be significantly correlated with that of PD-L1 in immune cells; in tumor cells, the expression of YKL-40 did not correlate with the expression of PD-L1 [1]. In melanoma, YKL-40 was demonstrated to upregulate PD-L1 expression indirectly and directly by increasing the production of PD-L1 in monocytes stimulated by IFN-gamma. Moreover, Ma et al. revealed that bispecific antibodies that simultaneously target YKL-40 and PD-1 have impressive synergistic antitumor effects, inducing CD8+, perforin+, and granzyme+ cytotoxic T cells and generating tumor cytotoxicity [60]. A synergistic effect of YKL-40 knockdown combined with anti-PD-L1 therapy has also been demonstrated in the murine model of diffuse large B-cell lymphoma (DLBCL). It was shown that treatment of mice with anti-PD-L1 therapy combined with YKL-40 knockout achieved significant inhibition of tumor growth compared to the separate use of anti-PD-L1 antibodies and YKL-40 knockout. Moreover, knockdown of YKL-40 enhances the proapoptotic effect of the anti-PD-L1 antibody in the mouse model of DLBCL [61].

GSEA results (Figure 3) suggest that high CHI3L1 expression is associated with biological processes involved in cytokine production (IL-2 and IL-6), mTORC1 and complement signaling, interferon-γ response, and inflammatory response, leading to an immunosuppressive effect in the tumor microenvironment. In the analysis performed by CAMOIP (Figure 4), CHI3L1 (YKL-40) was shown to have significant associations with various immune cells, proliferation rate, TGF-Beta response, lymphocyte infiltration signature score, and Th1/Th2 cell balance in the microenvironment of CRC. These findings suggest that YKL-40 may play a role in regulating the immune response and promoting tumor growth in colorectal cancer. The different types of immune cells, including M0, M1, M2 macrophages, and Tregs, play a crucial role in regulating the immune response in colorectal cancer [62,63,64]. M1 macrophages have a proinflammatory role and can help the immune system to attack cancer cells, while Tregs are known to suppress the immune response. On the other hand, M2 macrophages have a more suppressive role and can create an immune-suppressive microenvironment that allows cancer cells to escape detection by the immune system. The relationship between reduced YKL-40 expression and increased T-cell CD4 memory resting infiltration suggests that low CHI3L1 levels in colorectal cancer may be associated with a more favorable immune microenvironment for immunotherapy, as increased T-cell CD4 memory resting infiltration can enhance the immune system’s ability to attack cancer cells. On the other hand, the association between increased YKL-40 expression and increased lymphocyte infiltration signature score suggests that high YKL-40 levels in colorectal cancer may contribute to the infiltration of lymphocytes into the tumor microenvironment. However, the influence of CHI3L1 expression on the immunological properties of the CRC tumor microenvironment requires further investigation.

### 4.1. Correlation with Clinical Data

In the results obtained from tissue homogenates, we did not find any significant correlations between the concentrations of the studied molecules and clinical parameters such as TNM scale, stage, and grading. These results are not in line with previous studies and are probably associated with limitations of the present research, including the small number of patients. However, in the GEO analysis, we observed significantly higher expression of YKL-40 in the CRC relapse group in GEO dataset GDS4513 (Figure 2). According to literature data, both high tumor expression and elevated serum levels of YKL-40 are associated with poor outcomes in CRC patients, i.e., shorter overall survival and progression-free survival [65], which were not analyzed in our research. On the contrary, the association between YKL-40 expression and clinicopathological features such as tumor size, lymph node involvement, and histological grading has not yet been confirmed [1].

Peltonen et al. reported a correlation between MMP-8 tumor expression and its serum levels in CRC patients [66]. Increased serum levels of MMP-8 were reported to correlate with shorter survival, and inversely, lower MMP-8 levels were associated with a better prognosis. Elevated serum levels of MMP-8 were also described to be associated with distant metastasis, advanced primary tumor stage and grade [57,67], and high serum levels of CRP and several cytokines including IL-1ra, IL-7, and IL-8 [68].

In CRC, the expression of IL-17A was found to be significantly increased in patients with advanced disease [69]. However, IL-17 is also considered to play a protective role in CRC due to the positive correlation between its expression and early Dukes’ stage. This observation seems to be confirmed by a higher survival rate among patients with positive IL-17 expression compared to negative cases [70].

Similarly, the results obtained from tissue homogenates revealed no significant difference in the levels of studied molecules in individuals with obesity, diabetes, and smoking. Our results suggest no effect of comorbidities included in the analysis on YKL-40 expression in the colorectal cancer TME.

Further progress in research on the direct interactions of CHI3L1 expression with the expression and activity of PD-L1 due to the detailed study of PD-L1 in the tumor pathology and the use of the knowledge about PD-L1 not only in immunotherapy but also in dynamic phototherapy may contribute to overcoming resistance to immunotherapies, chemotherapy, and radiotherapy [71,72]. Due to ongoing studies using molecules targeting CHI3L1 expression such as small molecules targeting YKL-40 and chitosan, our high-dimensional analysis demonstrating associations between CHI3L1 expression and processes related to immunosuppression and the tumor microenvironment in colorectal cancer may contribute to a more thorough understanding and shape further research on the role of CHI3L1 in the pathology of colorectal cancer [73,74].

### 4.2. Study Limitation

Our study included a limited number of patients, which may be responsible for the lack of association between the levels of the investigated proteins and clinicopathological features of patients. Moreover, we are aware that the correlations reported in this study cannot confirm a direct association between the expression of the studied molecules. However, analysis of the correlations between the studied molecules and the use of GEO datasets and GSEA, as well as evaluation of their concentrations in tissue homogenates, sheds light on the association of YKL-40 expression with immune properties of the tumor microenvironment in CRC.

## 5. Conclusions

YKL-40 may play a pleiotropic role in mechanisms supporting tumor progression, which suggests that YKL-40 can be considered a promising therapeutic candidate. Diabetes, smoking, and obesity are not related to YKL-40 expression in colorectal cancer tissues. The associations of increased YKL-40 expression with appropriate cells in the tumor microenvironment and with the dysregulation of appropriate signaling pathways and biological processes demonstrated our GSEA support the significant role of YKL-40 in the process of formation of the immunosuppressive tumor microenvironment in CRC. The involvement of YKL-40 in the pathogenesis of CRC, as well as its potential as a molecule used as a component of targeted therapy in CRC, requires further research, with a particularly focus on the association of YKL-40 with CRC progression, depending on tumor pathogenetics, such as MSI status and the other molecular subtypes of CRC.

## Figures and Tables

**Table 1:** Characteristics of the study sample.

	Female	Male	
	20	28	48 (100%)
Age	63.06 ± 11.27	63.81 ± 8.61	63,47 ± 9.75
BMI	24.99 ± 5.06	26.75 ± 4.50	25.96 ± 4.78
Obesity			
Yes	3 (15%)	5 (17.86%)	8 (16.67%)
No	17 (85%)	23 (82.14%)	40 (83.33%)
Diabetes			
Yes	4 (20%)	10 (35.71%)	14 (29.17%)
No	16 (8%)	18 (64.29%)	34 (70.83%)
Nicotinism			
Yes	8 (40%)	9 (32.14%)	17 (35.42%)
No	12 (60%)	19 (67.86%)	31 (64.58%)
T parameter			
T1	0 (0%)	0 (0%)	0 (0%)
T2	7 (35%)	5 (17.86%)	12 (25%)
T3	10 (50%)	14 (50%)	24 (50%)
T4	3 (15%)	9 (32.14%)	12 (25%)
N parameter			
N0	9 (45%)	12 (42.86%)	21 (43.75%)
N1	8 (40%)	9 (32.14%)	17 (35.42%)
N2	3 (15%)	7 (25%)	10 (20.83%)
M parameter			
M0	18 (90%)	19 (67.86%)	37 (77.08%)
M1	2 (10%)	9 (32.14%)	11 (22.92%)
TNM stage			
I	6 (30%)	4 (14.29%)	10 (20.83%)
II	3 (15%)	7 (25.00%)	10 (20.83%)
III	9 (45%)	8 (28.57%)	17 (35.42%)
IV	2 (10%)	9 (32.14%)	11 (22.92%)
Grading			
G1	1 (5%)	0 (0%)	1 (2.08%)
G2	19 (95%)	28 (100%)	47 (97.92%)
G3	0(0%)	0 (0%)	0 (0%)

**Table 2:** Levels of YKL-40, PD-L1, MMP-8, IL-17, and IL-23 proteins in tumor and margin presented as log-transformed ng/mg of protein. Paired Student’s *t*-test.

	Tumor		Margin		*p*
	Mean	SD	Mean	SD	
log YKL-40	1.20	0.27	0.71	0.22	<0.0001
log PD-L1	0.69	0.27	0.52	0.22	0.001
log MMP-8	1.58	0.46	0.96	0.37	<0.0001
log IL-17	−1.74	0.26	−1.95	0.29	<0.0001

**Table 3:** Correlations between the YKL-40 levels and the examined molecules. R—Pearson’s correlation coefficient.

Pair of Variables	R	*p*
Tumor log YKL-40 and tumor log PD-L1	0.32	0.044
Tumor log YKL-40 and tumor log MMP-8	0.71	<0.0001
Tumor log YKL-40 and tumor log IL-17	0.36	0.026
Tumor log YKL-40 and margin log YKL-40	0.32	0.032
Margin log YKL-40 and margin log PD-L1	0.37	0.022
Margin log YKL-40 and margin log IL-17	0.49	<0.0001
Margin log YKL-40 and margin log MMP-8	0.70	<0.0001
Margin log YKL-40 and tumor log IL-17	0.49	0.022
Margin log YKL-40 and tumor PD-L1	0.37	0.022
Tumor log PD-L1 and tumor IL-17	0.75	<0.001

**Table 4:** Correlations between the YKL-40 levels and the examined molecules. R—Pearson’s correlation coefficient.

	Mean	SD	Mean	SD	*p*
log YKL-40_tumor	Diabetes		no Diabetes		
	1.22	0.31	1.2	0.24	0.79
	obesity		no obesity		
	1.22	0.43	1.2	0.25	0.86
